# An integrative pan-cancer analysis of molecular characteristics and oncogenic role of mitochondrial creatine kinase 1A (CKMT1A) in human tumors

**DOI:** 10.1038/s41598-022-14346-z

**Published:** 2022-06-15

**Authors:** Mengjie Yang, Shuna Liu, Yue Xiong, Jingxin Zhao, Wenbin Deng

**Affiliations:** 1 School of Pharmaceutical Sciences (Shenzhen), Sun Yat-Sen University, No. 66, Gongchang Road, Guangming District, Shenzhen, 518107 China; 2 Jiangxi Deshang Pharmaceutical Co., Ltd., Zhangshu, China

**Keywords:** Cancer genetics, Cancer, Computational biology and bioinformatics

## Abstract

In recent years, several studies have suggested that mitochondrial creatine kinase 1A (CKMT1A) plays a key role in various cancer types. However, there is still a lack of systematic understanding of the contribution of CKMT1A in different types of cancer. Therefore, this study aims to explore the potential role of CKMT1A in human tumors. Firstly, we evaluated the expression level of CKMT1A in 33 types of tumors. Secondly, we used the GEPIA2 and Kaplan–Meier plotter to explore the relationship between CKMT1A expression and survival prognosis. Furthermore, the genetic alterations of CKMT1A were analyzed by the cBioPortal web. In addition, we performed immune infiltration analysis and gene enrichment pathway analysis. CKMT1A was highly expressed in most types of cancers and there was a significant correlation between CKMT1A expression and the prognosis of patients for certain tumors. Non-Small Cell Lung Cancer cases with altered CKMT1A showed a poorer overall survival. CKMT1A expression was negatively correlated with the infiltration of cancer-associated fibroblasts in most tumors. We also found that its expression was negatively associated with CD8^+^ T-cell infiltration in several tumors. Furthermore, enrichment analysis revealed that “Glycolysis/ Gluconeogenesis” and “metabolic pathways” functions were involved in the functional mechanism of CKMT1A. Taken together, our studies will provide a relatively clear and integrative understanding of the role of CKMT1A across different tumors. All these findings will lay a solid foundation for further molecular assays of CKMT1A in tumorigenesis and provide the rationale for developing novel therapeutic strategies.

## Introduction

Over the past few decades, cancer has long been a focus of scientific research since it is one of the leading causes of morbidity and mortality worldwide^1,2^. Cancer is a complex and multifaceted process involving many oncogenes as well as environmental risk factors^3^. Influenced by the tumor microenvironment, one gene may play different roles in different tumors. In addition, studying a gene in a particular tumor alone is not sufficient to understand its function. Therefore, it is very important to conduct a pan-cancer analysis of any gene and verify its clinical role and potential molecular mechanisms^4^. The public and free databases, such as TCGA and GEO database, provides us with convenient access to conduct pan-cancer analysis^5–7^.

Mitochondrial creatine kinase 1A (CKMT1A) is a mitochondrial protein that exists on the outer surface of the inner membrane of mitochondria. Its main function is to facilitate the transfer of phosphocreatine (PCr) energy across mitochondria by transferring phosphate groups from mitochondrial ATP to creatine (Cr)^8^. Many studies have shown that CKMT1A plays different roles in many different tumors. It is highly expressed in liver cancer, lung cancer and breast cancer tissue and promotes the malignant growth of tumor cells, which is associated with poor prognosis of patients^9–11^. However, in oral cancer, prostate cancer and glioma^12–14^, CKMT1A expression level in tumor tissues is lower than that in the normal tissues. In addition, it also reduces the sensitivity of nasopharyngeal carcinoma to radiotherapy, which can be a target to increase the efficacy of radiotherapy^15^. Considering the specific mechanism by which CKMT1A regulates cell function in cancer is not well understood, we seek to perform a pan-cancer analysis to fully understand its function in various tumor types.

In this study, we conducted a pan-cancer analysis of CKMT1A based on the data of the TCGA project and GEO database. We analyzed gene expression, survival prognosis, genetic alteration, immune infiltration, and cellular pathway, to explore the potential mechanisms of CKMT1A across different cancers.

## Materials and methods

### Gene expression analysis

To obtain the expression profile of CKMT1A in cancer, we generated the box plots of the expression difference of CKMT1A between tumor and adjacent normal tissues for the different tumors or specific tumor subtypes in the TCGA project by using the “Exploration—Gene_DE” module of TIMER2 (tumor immune estimation resource, version 2) web (http://timer.comp-genomics.org/). For those without normal tissues matched as controls in TIMER2, the “Expression Analysis—Expression DIY—Box Plot” function of the GEPIA2^16^ (Gene Expression Profiling Interactive Analysis, version 2) web tool (http://gepia2.cancer-pku.cn/#analysis) was used to obtain box plots of the expression level difference of CKMT1A between the tumor and corresponding normal tissues of the GTEx (Genotype-Tissue Expression) database, and the settings of *P* value cutoff = 0.01, log_2_FC (Fold Change) cutoff = 1. And also, we used the “Expression Analysis—Expression DIY—Stage Plot” function of GEPIA2 to get violin plots of the CKMT1A expression in different pathological stages. The UALCAN portal (http://ualcan.path.uab.edu/analysis-prot.html) is an online tool for analyzing protein expression analysis option using data from Clinical Proteomic Tumor Analysis Consortium (CPTAC) Confirmatory/Discovery dataset^17^. Therefore, we also performed the expression level of CKMT1A between primary tumor and normal tissues in available cancer types of CPTAC, such as UCEC (Uterine corpus endometrial carcinoma), clear cell RCC (Renal cell carcinoma), ovarian cancer, colon cancer, lung adenocarcinoma and breast cancer.

### Survival prognosis analysis

We used the “Expression Analysis—Survival analysis—Survival Map” function of GEPIA2 to obtain the OS (Overall survival) and DFS (Disease-free survival) significance map data of CKMT1A in all TCGA tumors. The median of CKMT1A expression was used to divide all cases into the high-expression group and low-expression group. The log-rank test was used to test the statistical significance, and the Kaplan–Meier survival plots of OS and DFS were also determined using the “Expression Analysis—Survival analysis” function of GEPIA2. In addition, we also used the Kaplan–Meier plotter tool (https://kmplot.com/analysis/index.php?p=background) to analyze the relationship between the prognosis of patients and CKMT1A expression in breast cancer, ovarian cancer, lung cancer and gastric cancer^18^. And the “Auto select best cutoff” function was used to divide all patients into two groups for survival analysis.

### Genetic alteration analysis

We logged into the cBioPortal web (https://www.cbioportal.org/)^19,20^, and then chose the “TCGA Pan Cancer Atlas Studies” in the “Quick select” section. After entering the interface, we entered “CKMT1A” into the Query Box and obtained the results of the genetic alteration characteristics of CKMT1A. The results of the alteration frequency, mutation type and CNA (Copy number alteration) in all TCGA tumors were observed in the “Cancer Types Summary—Cancer Type” module. In the module of “mutations”, the mutated site in the protein structure, the PTM (Post Translational Modification) site and the 3D (Three-dimensional) structure information of CKMT1A were displayed. We also used the “Comparison” module to analyze the survival progression differences (overall, progression-free, and disease-free survival) for the TCGA cancer cases with or without CKMT1A genetic alteration. Kaplan–Meier survival plots were generated and statistical significance by log-rank P-value was computed as well.

### Immune infiltration analysis

The association between CKMT1A expression and immune infiltrates in all TCGA tumors was explored through the “Exploration—Immune-Gene” module of the TIMER2. We selected the CD8^ +^ T-cells and cancer-associated fibroblasts for analysis. The immune infiltrations were estimated by using the TIMER, CIBERSORT, CIBERSORT-ABS, QUANTISEQ, XCELL, MCPCOUNTER and EPIC algorithms. The P-values and partial correlation (Cor) values were obtained by Spearman's rank correlation test after purity-adjusted. And all data were displayed as a heatmap and scatter plot.

### CKMT1A‑related gene enrichment analysis

We searched the CKMT1A on the STRING website (https://string-db.org/), with the parameters setting was that the meaning of network edges was “confidence”, the minimum required interaction score was “medium confidence (0.4)”, and the max number of interactors to show was “no more than 50 interactors” in the 1st shell, and finally we obtained 50 proteins potentially interacted with CKMT1A. In addition, the top 100 CKMT1A-correlated targeting genes were obtained by using the “Expression Analysis—Similar Gene Detection” function of GEPIA2 based on the datasets of all TCGA tumors. The Pearson correlation analysis of CKMT1A and selected genes was performed by using the “Expression Analysis—correlation analysis” function of GEPIA2. Moreover, the heatmap of selected genes was obtained from the “Exploration—Gene_Corr” module of TIMER2, and the partial correlation (Cor) and P-value were obtained by Spearman's rank correlation test after purity-adjusted.

To perform intersection analysis of CKMT1A-binding and interacted genes, a web Venn diagram tool (http://bioinformatics.psb.ugent.be/webtools/Venn/) was applied. Moreover, we used these two datasets to perform Kyoto Encyclopedia of Genes and Genomes (KEGG) and Gene Ontology (GO) pathway analysis^21^. In brief, we uploaded the gene lists to KOBAS in the "enrichment—Gene list enrichment" module and then obtained the data of the functional annotation chart (http://kobas.cbi.pku.edu.cn/)^22^. The KEGG enriched pathways were finally visualized in the “Gene list enrichment visualization” module.

### Accordance statements

The study was performed in accordance with relevant guidelines and regulations of openly available database.

### Ethics approval and consent to participate

Not applicable. TCGA and GEO belong to public databases. The patients involved in the database have obtained ethical approval. Our study was based on open source data, so there were no ethical issues and other conflicts of interest.

## Results

### The data of gene expression analysis

**Figure 1:**
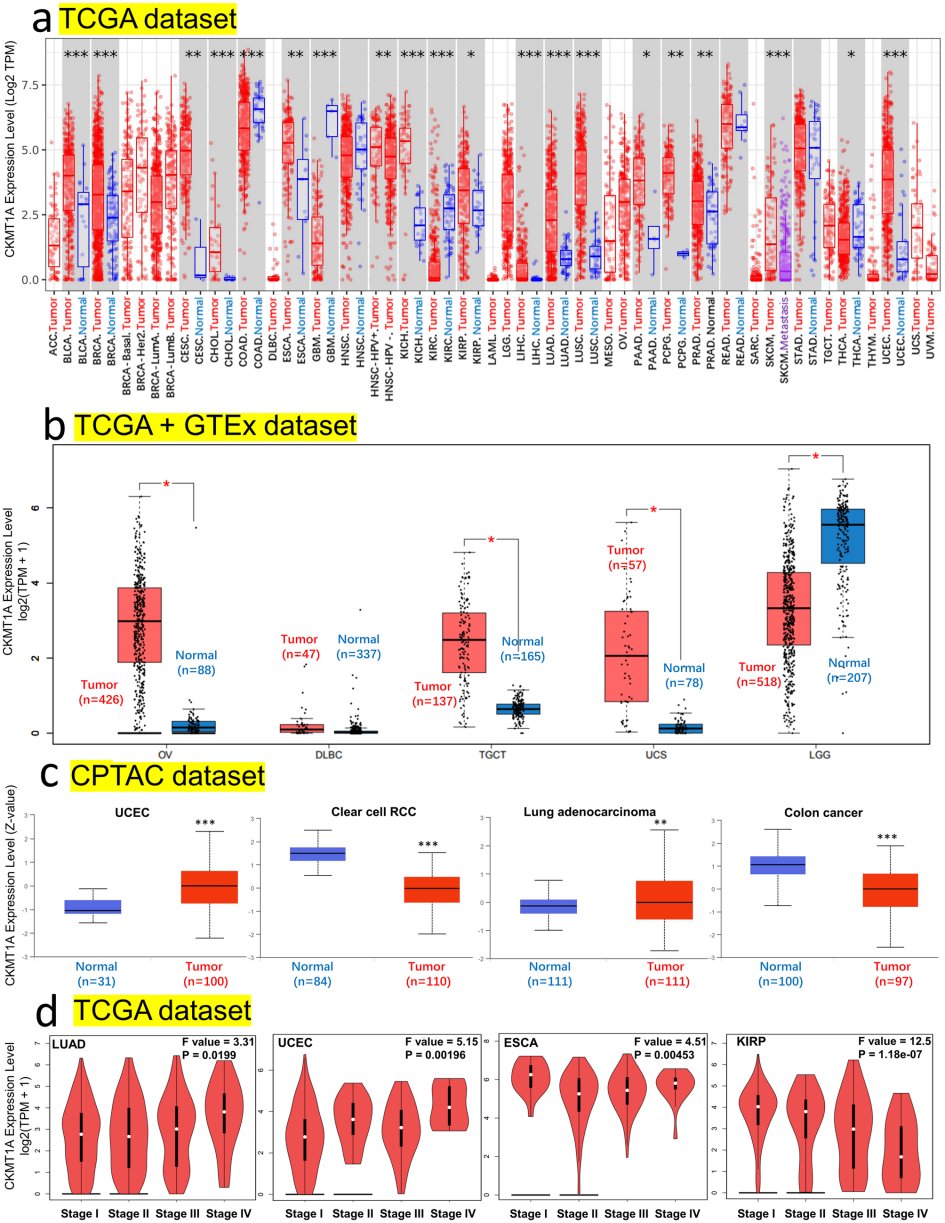
The CKMT1A Expression level in different tumors and pathological stages. (**a**) The expression of CKMT1A in different cancers or specific cancer subtypes in TCGA dataset. (**b**) The expression of CKMT1A in OV, DLBC, TGCT, UCS and LGG in the TCGA project were compared with the corresponding normal tissues of the GTEx database. (**c**) Based on the CPTAC dataset, the expression level of CKMT1A between normal tissue and primary tissue of UCEC, clear cell RCC, lung adenocarcinoma and colon cancer were also analyzed. (**d**) According to the TCGA data, the expression levels of the CKMT1A were analyzed by the main pathological stages (stage I, stage II, stage III, and stage IV) of LUAD, UCEC, ESCA, and KIRP. Log2 (TPM + 1) was utilized for log-scale. *P < 0.05; **P < 0.01; ***P < 0.001.

In this study, we aimed to explore the role of human CKMT1A (NM_001015001.2 for mRNA or NP_001015001.1 for protein) in cancer. To determine the expression level of CKMT1A, we used the TIMER2 web tool to analyze the CKMT1A in various cancer types of TCGA. As shown in Fig. 1a, the expression of CKMT1A in the tumor tissues of BLCA (Bladder urothelial carcinoma) (*P* < 0.001) , BRCA (Breast invasive carcinoma) (*P* < 0.001), CESC (Cervical squamous cell carcinoma and endocervical adenocarcinoma) (*P* < 0.01), CHOL (Cholangio carcinoma) (*P* < 0.001), ESCA (Esophageal carcinoma) (*P* < 0.01), KICH (Kidney chromophobe) (*P* < 0.001), KIRP (Kidney renal papillary cell carcinoma) (*P* < 0.05), LIHC (Liver hepatocellular carcinoma) (*P* < 0.001), LUAD (Lung adenocarcinoma) (*P* < 0.001), LUSC (Lung squamous cell carcinoma) (*P* < 0.001), PAAD (Pancreatic adenocarcinoma) (*P* < 0.05), PCPG (Pheochromocytoma and Paraganglioma) (*P* < 0.01), PRAD (Prostate adenocarcinoma) (*P* < 0.01), UCEC (Uterine Corpus Endometrial Carcinoma) (*P* < 0.001) was significantly higher than the corresponding normal control tissues. And the expression of CKMT1A in the tumor tissues of COAD (Colon adenocarcinoma) (*P* < 0.001), GBM (Glioblastoma multiforme) (*P* < 0.001), KIRC (Kidney renal clear cell carcinoma) (*P* < 0.001), THCA (Thyroid carcinoma) (*P* < 0.05) was significantly lower than the corresponding control tissues.

Because some kinds of tumor types have no corresponding control tissues in TIMER2 database, we analyzed the expression difference of CKMT1A between normal and tumor tissues by using the normal tissue of the GTEx dataset as controls in GEPIA2. As shown in Fig. 1b, the CKMT1A expression level was significantly higher in tumor tissues than in the corresponding controls in OV (Ovarian serous cystadenocarcinoma), TGCT (Testicular germ cell tumors) and UCS (Uterine carcinosarcoma) (all *P* < 0.05). The CKMT1A expression level was significantly lower in tumor tissues of LGG (Brain lower grade glioma) (Fig. 1b, *P* < 0.05). But we didn’t obtain a significant difference in DLBC (Lymphoid Neoplasm Diffuse Large B-cell Lymphoma).

According to the CPTAC datasets, the expression level of CKMT1A in the tissues of UCEC (*P* < 0.001) and LUAD (*P* < 0.01) was also significantly higher than in normal tissues (Fig. 1c). The results showed lower expression of CKMT1A in the primary tissues of clear cell RCC (*P* < 0.001) and colon cancer (*P* < 0.001) than in normal tissues (Fig. 1c), but there was no significant difference in ovarian cancer and breast cancer (Additional file 1 Fig. S1a). The correlation between the expression level of CKMT1A and the pathological stages of tumors was explored through the function of “Expression Analysis—Expression DIY—Stage Plot” in GEPIA2. We observed a positive correlation between CKMT1A expression and cancer pathological stages in LUAD and UCEC (Fig. 1d, all *P* < 0.05), and a negative correlation in ESCA and KIRP (Fig. 1d, all *P* < 0.05), the violin plots of other cancers were show in Fig. S1b (Additional file 1 Fig. S1b).

### The results of survival analysis

**Figure 2:**
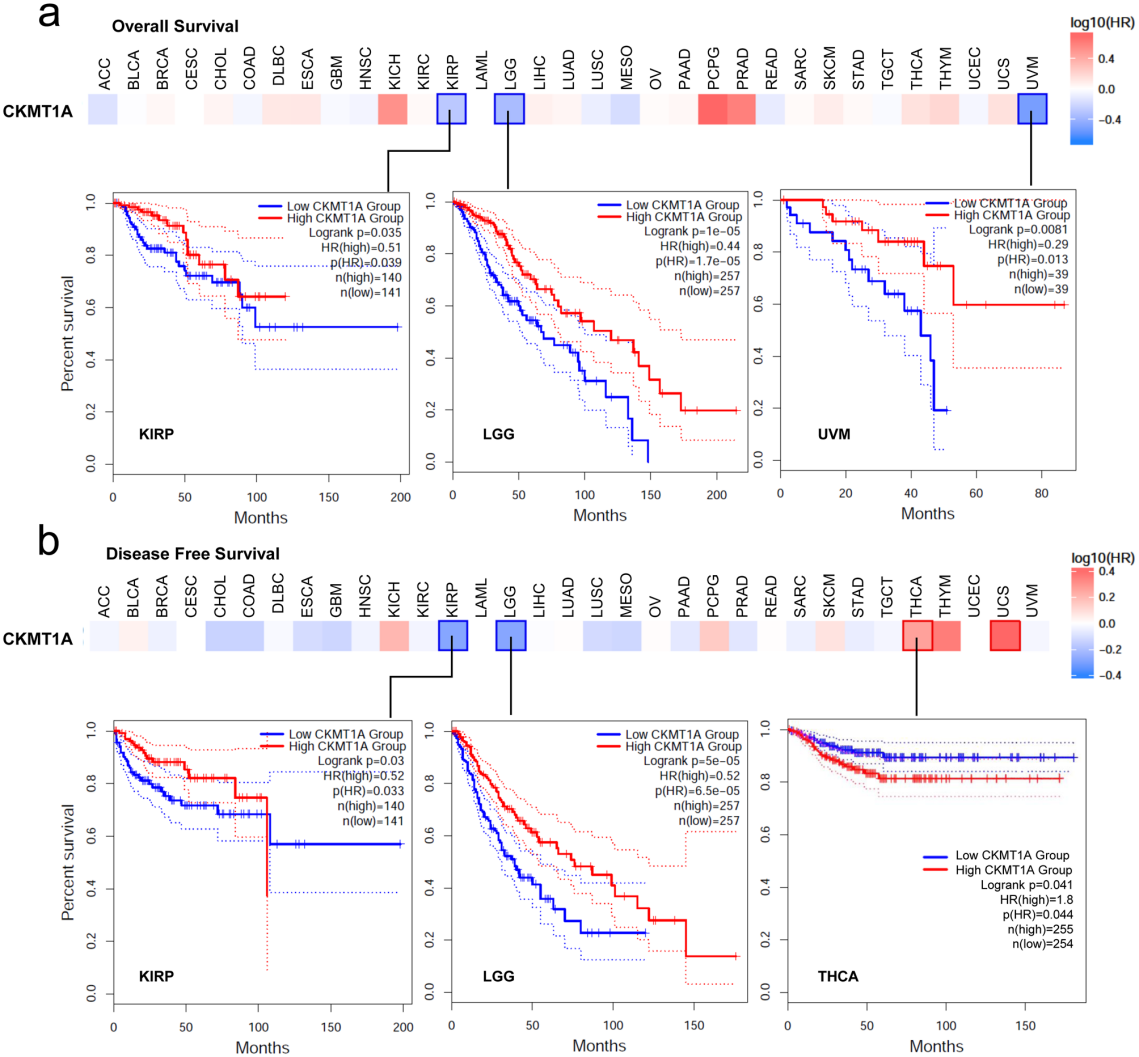
The survival map and Kaplan–Meier curves between the expression level of CKMT1A and survival prognosis of cancers in TCGA. (**a**) The correlation between the expression level of CKMT1A and overall survival in KIRP, LGG and UVM. (**b**) The correlation between the expression level of CKMT1A and disease-free survival in KIRP, LGG and THCA.

According to the expression median levels of CKMT1A, we divided the cancer cases into high-expression and low-expression groups. And using the datasets of TCGA and GEO, the correlations of CKMT1A expression with the prognosis of patients across different tumors were investigated. As shown in Fig. 2a, high expression of CKMT1A was associated with a good prognosis of overall survival (OS) for cancers of KIRP (*P* = 0.035), LGG (*P* < 0.001) and UVM (*P* = 0.0081) in the TCGA project. For disease-free survival (DFS) analysis, the results showed that high CKMT1A expression was correlated with good prognosis in KIRP (*P* = 0.03) and LGG (*P* < 0.001) of TCGA datasets but was correlated with poor prognosis for THCA (Fig. 2b, *P* = 0.041. According to the results from the Kaplan–Meier plotter tool, we found a correlation between high expression CKMT1A and poor FP (First progression) (*P* < 0.001), OS (*P* < 0.001) and PPS (Post-progression survival) (*P* = 0.01) prognosis for lung cancer and poor RFS (Relapse-free survival), OS, DMFS (Distant metastasis-free survival) and PPS prognosis (all *P* < 0.05) for breast cancer cases (Additional file 1 Fig. S2). However, a low CKMT1A expression level was associated with poor OS (*P* = 0.0019), FP (*P* < 0.001), and PPS (*P* = 0.018) prognosis for gastric cancer and poor RFS (P = 0.018) prognosis of liver cancer (Additional file 1 Fig. S3). In contrast, a high expression level of CKMT1A was related to a poor PPS (*P* = 0.0092) prognosis for ovarian cancer (Additional file 1 Fig. S2). We failed to detect a correlation between CKMT1A expression and the PPS, OS prognosis of ovarian, and the PFS (Progress-free survival), DSS (Disease-specific survival) and OS prognosis of liver cancer (Additional file 1 Fig. S2/S3, all *P* > 0.05).

### The data of genetic alteration

**Figure 3:**
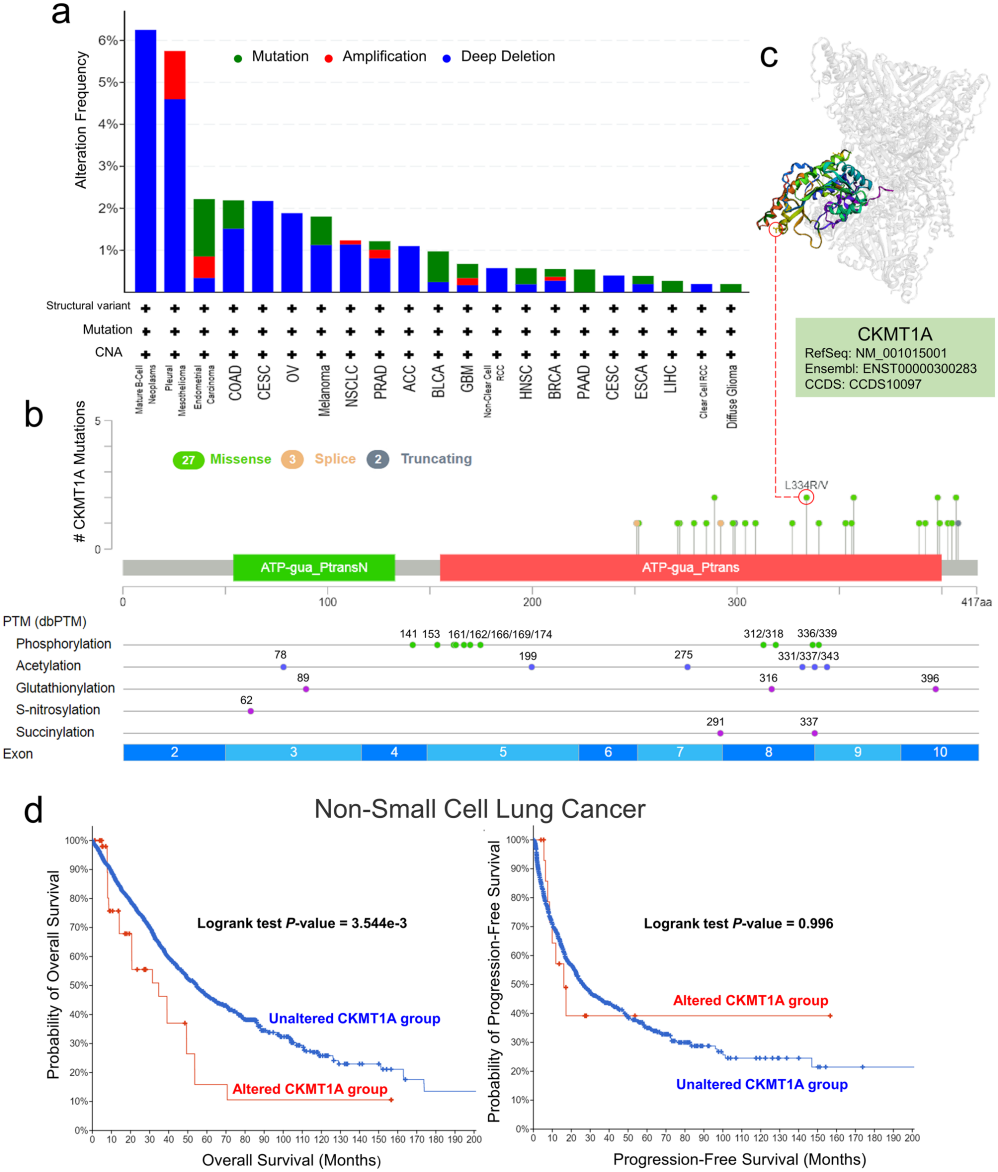
Mutation feature of CKMT1A in different tumors of TCGA. (**a**) The alteration frequency with mutation type of CKMT1A for the TCGA tumors were analyzed by cBioPortal tool. (**b**) The mutation site and PTM of CKMT1A were displayed. (**c**) The mutation site L334R/V in the 3D structure of CKMT1A. (**d**) The correlation between mutation status and overall survival, progression-free survival of Non-Small Cell Lung Cancer were also analyzed.

The genetic alteration of CKMT1A was explored across different cancer subtypes in TCGA dataset. As presented in Fig. 3a, mutations, amplifications and deep deletions were the main types of CKMT1A alterations. Deep deletion was the most common type of alteration in Mature B-Cell Neoplasms, Pleural Mesothelioma, CESC, OV, ACC and Non-Clear Cell RCC. Moreover, the CKMT1A alterations of the “amplification” type of CNA were mostly found in Pleural Mesothelioma and Endometrial Carcinoma cases, with the frequency of ~ 1%. The “mutations” type of CKMT1A accounted for the vast majority of alterations in Endometrial Carcinoma cases (Fig. 3a). The types, sites of CKMT1A genetic alteration and PTM sites were presented in Fig. 3b. Detailed analysis showed that the main type of genetic alteration for CKMT1A was missense mutation. For example, L334R/V alteration was a missense mutation in the ATP-gua_Ptrans domain and was able to change the 334 site of CKMT1A protein, thus might affect the function of the protein. And the L334 site was displayed in the 3D structure of CKMT1A (Fig. 3c). According to the results of PTM in Fig. 3b, we could observe the phosphorylation sites, the acetylation sites and other PTM sites of CKMT1A. Phosphorylation modification was the main type of PTM in CKMT1A, with a total of 11 sites, followed by acetylation which had 6 sites (Fig. 3b). Furthermore, we explored the association between genetic alteration of CKMT1A and the clinical survival prognosis in different types of cancers. The result of Fig. 3d showed that the cases who had altered CKMT1A appeared more bad prognosis in overall survival (*P* = 3.544e−3), but not progression-free survival (*P* = 0.996), compared with cases without CKMT1A alteration in Non-Small Cell Lung Cancer.

### Immune infiltration analysis data

**Figure 4:**
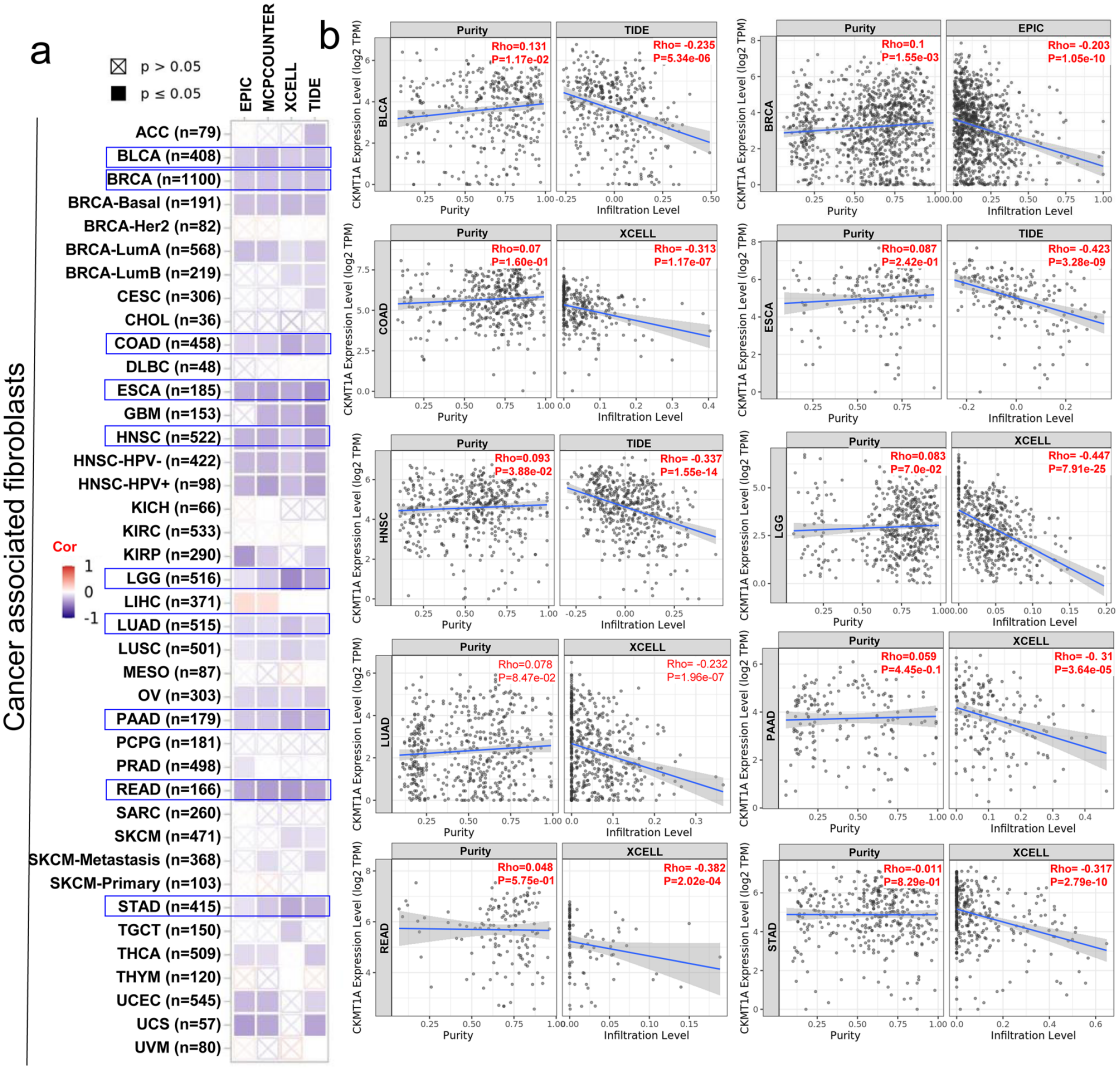
Correlation analysis between the expression of CKMT1A and immune infiltration of cancer-associated fibroblasts. (**a**) The correlation map between CKMT1A expression level and the infiltration level of cancer-associated fibroblasts by EPIC, MCPCOUNTER, XCELL and TIDE algorithms across all types of cancer in TCGA. (**b**) The relationship of CKMT1A and infiltration level of cancer-associated fibroblasts in BLCA, BRCA, COAD, ESCA, HNSC, LGG, LUAD, PAAD, READ and STAD. Rho, The Value of Spearman’s correlation.

Infiltrating immune cells are the main and important components of the tumor microenvironment, which are frequently associated with tumor behavior, drug resistance and patient outcomes^23,24^. Accumulating evidence has demonstrated that cancer-associated fibroblasts (CAFs) are the predominant cell type within the tumor stroma and mainly facilitate tumor cells immune escape by modulating the infiltrating immune cells^25,26^. Therefore, in this study, we used the TIMER, XCELL, QUANTISEQ, MCPCOUNTER, CIBERSORT, CIBERSORT-ABS and EPIC algorithms to investigate the correlation between the infiltration level of diverse immune cells and CKMT1A expression across various cancer types of TCGA in TIMER 2.0. After systematic analyses, we found that the estimated infiltration value of CAFs was significantly negatively correlated with the expression of CKMT1A in most TCGA tumor types based on all or most algorithms, but not in LIHC (Fig. 4a). The scatterplot data of the selected tumors produced using one algorithm were presented in Fig. 4b. For example, the expression level of CKMT1A in LGG was negatively correlated with the infiltration level of cancer-associated fibroblasts (Fig. 4b, cor =  − 0.447, *P* = 7.91e−25) based on the XCELL algorithm. We also found a statistically negative correlation between the immune infiltration of CD8^+^ T-cells and CKMT1A expression in the tumors of MESO, STAD, THYM, UCEC and SKCM-Primary based most algorithms (cor =  − 0.441, *P* = 8.35e−07 based on the EPIC algorithm for THYM) (Additional file 1 Fig. S4).

### The data of enrichment analysis of CKMT1A

**Figure 5:**
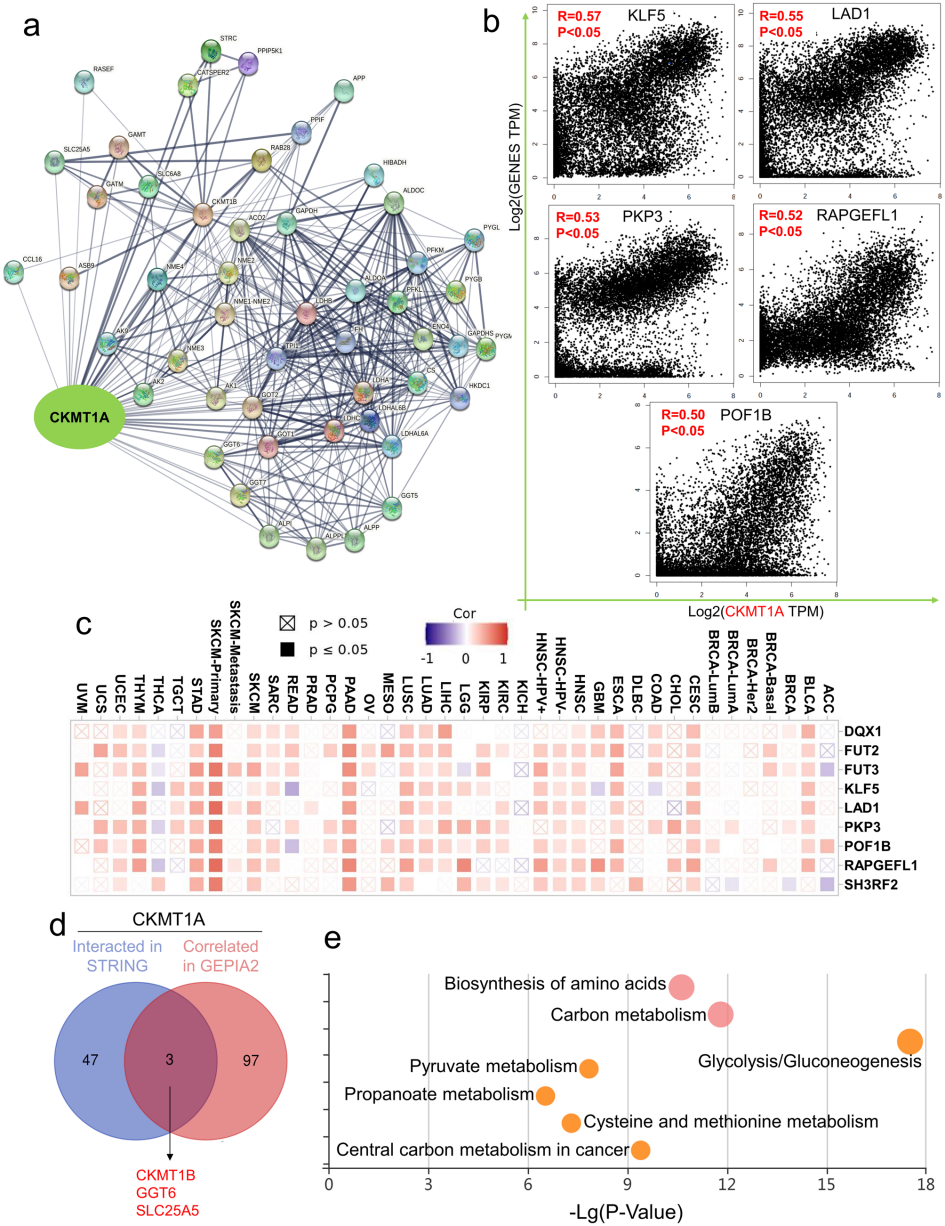
The enrichment analysis of CKMT1A-related genes. (**a**) The network of 50 CKMT1A-interacting proteins in the STRING tool. (**b**) The top 100 CKMT1A-correlated genes were obtained in TCGA projects and the expression correlation between CKMT1A and selected 5 genes, including KLF5, LAD1, PKP3, POF1B, and RAPGEFL1 were analyzed by GEPIA2 approach. (**c**) The heatmap of correlation between CKMT1A and selected top 10 genes, CKMT1B excluded, in different cancer types. (**d**) The Venn diagram of the CKMT1A interacted in STRING and correlated genes in GEPIA2. (**e**) The KEGG pathway analysis of CKMT1A-interacted and the correlated genes.

To further explore the molecular function of the CKMT1A gene in cancer, we attempted to screen out the CKMT1A correlated genes and CKMT1A-interacting proteins for subsequent pathway enrichment analyses. We obtained 50 potential CKMT1A interacting proteins using the STRING tool and their interaction network was shown in Fig. 5a. Then we used the “Similar Gene Detection” function of GEPIA2 to obtain the top 100 genes correlated with CKMT1A with all tumor expression data of TCGA included. The top 100 correlated genes and 50 potential interacting proteins were listed in Additional file 2. As CKMT1B is another isoform of CKMT1A and their functions are similar, we didn’t explore their relationship in this study. The scatterplots of the top 5 correlated genes excluded CKMT1B were shown in Fig. 5b, CKMT1A expression was significantly positively correlated with KLF5 (Kruppel like factor 5) (R = 0.57), LAD1 (ladinin 1) (R = 0.55), PKP3 (plakophilin 3) (R = 0.53), RAPGEFL1(Rap guanine nucleotide exchange factor like 1) (R = 0.52) and POF1B (POF1B actin binding protein) (R = 0.50) genes (all *P* < 0.001). The corresponding heatmap data also showed a positive correlation between CKMT1A and the top 10 correlated genes (excluded CKMT1B) in most cancer types (Fig. 5c). After intersection analysis of these two groups, we discovered three common members (CKMT1B, GGT6, SLC25A5) in the two datasets (Fig. 5d). In addition, we performed KEGG and GO pathway enrichment analysis with a combined dataset of the above two datasets. The KEGG pathway suggested that “Glycolysis/Gluconeogenesis”, “Carbon metabolism”, “Biosynthesis of amino acids” and “metabolic pathways” might be involved in the effect of CKMT1A on tumor pathogenesis (Fig. 5e, Additional file 3). The GO enrichment analysis indicated that most of these genes were significantly connected with the pathways or cellular biology of protein binding and glycolytic process (Additional file 4).

## Discussion

Emerging studies have shown that CKMT1A may participate in a series of cellular biological in the occurrence and development of human cancers such as cell proliferation, cell migration and apoptosis^11–13,15^. However, its role in most types of cancer remains controversial and has not been well defined. Whether CKMT1A plays a common pathogenic role in molecular mechanisms of different tumors remains unproven and requires further investigation. In this study, we performed a series of bioinformatics analysis of CKMT1A regarding its expression, gene function, genetic alteration, immune infiltration and survival prognostic in 33 cancer types based on TCGA, GEO and CPTAC databases. Our findings provided a relatively comprehensive and intuitive map of CKMT1A for future research in cancer.

In our study, we found that CKMT1A was highly expressed in most cancer, but was lowly expressed in COAD, GBM, KIRC, THCA and LGG compared to corresponding control tissues. According to previous reports, CKMT1A expression levels were increased in liver cancer, lung cancer and breast cancer cells^9–11^, and this was consistent with our findings. However, in oral cancer, prostate cancer and glioma, the expression level of CKMT1A was lower in tumor tissues than that in normal tissues^12–14^. For Head and Neck squamous cell (HNSC) in our study, we also found CKMT1A in tumors was low but with no statistical significance. The different expression patterns of CKMT1A in various tumors implied it seemed to have opposite effects on the biological function of cancer. Further studies are needed to find the different mechanisms. One previous study demonstrated that High CKMT1A expression in hepatocellular carcinoma denoted a poor prognosis with highly malignant potential^10^. Based on the datasets of the Kaplan–Meier plotter covering liver cancer cases in GEO data, we found an opposite result, namely lower CKMT1A expression relates to poorer RFP (*P* < 0.05) (Additional file 1 Fig. S3). The inconsistencies might be due to the evaluation of CKMT1A expression from serum samples in their study, as well as different endpoints for survival analysis. Ming Li (2021) found that a high level of CKMT1A was significantly correlated with the high pathological grade of lung cancer^11^. Here, our study supported this finding and also proved that higher expression of CKMT1A was related to the poor prognosis of patients in FS, OS and PPS (all *P* < 0.05, Additional file 1 Fig. S2). CKMT1A exerts different functions because cancer cells have different genetic backgrounds and are regulated by multiple factors and the combined effect of microenvironment^27,28^. We noticed that the results of survival analysis in Fig. S2 and Fig. S3 were different from Fig. 2. Differences in data sources, tumor classification and endpoints of survival analysis between the two databases may account for the different results. Consequently, larger studies which have more sample sizes and more factors are required to confirm the role of CKMT1A in cancer.

It is widely believed that cancers are triggered by genetic mutations, which biologically strengthen cancer cells to grow more rapidly than normal ones thus influencing the progression of cancer^29,30^. In our study, we evaluated the mutation patterns of CKMT1A in different tumors of TCGA. We confirmed that missense mutation was the predominant type of genetic alteration for CKMT1A (Fig. 3a). Furthermore, we explored the association between genetic alteration of CKMT1A and the clinical survival prognosis of lung cancer. The result showed that altered CKMT1A led to a more bad prognosis in overall survival. It is well known that PTMs may affect the function of transcribed gene products. We also displayed the potential PTMs type and site of CKMT1A in cancer. In breast cancer, oncogenic HER2 induced phosphorylation of CKMT1A and then stabilized CKMT1A to promote cancer cell proliferation^31^. Additional experiments are required to further evaluate the potential role of other CKMT1A phosphorylation sites in tumorigenesis.

The complex tumor microenvironment (TME) consisting of tumor cells and infiltrating immune cells plays a pivotal role in the development of tumors. CAFs are the predominant cell type within the tumor stroma and they maintain a favorable microenvironment for tumor cell proliferation^25^. We observed a statistically negative correlation between CKMT1A expression and the infiltration level of CAFs in most cancer. Such findings would give us a better clue that CKMT1A regulates the tumor microenvironment through altering the CAFs infiltration and might affect the therapy response of cancer. The host CD8^+^ T cells play an important role in the occurrence, development, and metastasis of tumors, its dysfunctions in tumors are associated with a poor clinical response^32^. In this article, we also demonstrated the negative association between CKMT1A expression and immune infiltration level of CD8^+^ T-cells in MESO, STAD, THYM, UCEC and SKCM-Primary. Due to these, CKMT1A is associated with immune infiltration and can be a potential prognostic biomarker for some certain tumors.

Cancer is a metabolic disease, and the function of CKMT1A in mitochondria plays an important role in the energy demand transformation process^8,12^. CKMT1A helps maintain energy conversion, and protects cells from death by preventing stressful situations such as hypoxia^11,15^. Furthermore, we integrated the information on CKMT1A-interacting proteins and CKMT1A-related genes for enrichment analyses and identified the potential impact of “metabolic pathways” and “Glycolysis/Gluconeogenesis” in the etiology or pathogenesis of cancers. Many new cancer therapeutic targets might be identified among the metabolic process in which CKMT1A is involved and cancer researches about them are needed in the future.

Our study also had some limitations. We explored the potential role of CKMT1A in human tumors through publicly available online databases but lack of our own data or experiment validation. And for some genes, RNA expression not always predictive of protein expression. Our research results were based on the TCGA database which always focuses on RNA expression. Therefore, more studies on CKMT1A protein expression and experiment validation in cancers are needed.

Taken together, our pan-cancer analysis of CKMT1A provided a comprehensive understanding of CKMT1A in gene expression, genetic alteration, clinical prognosis, immune cell infiltration and pathway analysis. All these findings will lay a solid foundation for further molecular assays of CKMT1A in tumorigenesis and provide the rationale for developing novel therapeutic strategies.

## Supplementary Information

Supplementary Information 1.

Supplementary Information 2.

Supplementary Information 3.

Supplementary Information 4.

## Data Availability

Our experimental datasets were available from the corresponding authors upon reasonable request. The datasets for this study can be found in the TCGA (https://www.cancer.gov/tcga), GEO (https://www.ncbi.nlm.nih.gov/geo/) and GTEx (http://commonfund.nih.gov/GTEx/).

## Abbreviations

ACC: Adrenocortical carcinoma
BLCA: Bladder urothelial carcinoma
BRCA: Breast invasive carcinoma
CESC: Cervical squamous cell carcinoma and endocervical adenocarcinoma
CHOL: Cholangio carcinoma
COAD: Colon adenocarcinoma
DLBC: Lymphoid neoplasm diffuse large B-cell lymphoma
ESCA: Esophageal carcinoma
GBM: Glioblastoma multiforme
HNSC: Head and neck squamous cell carcinoma
KICH: Kidney chromophobe
KIRC: Kidney renal clear cell carcinoma
KIRP: Kidney renal papillary cell carcinoma
LAML: Acute myeloid leukemia
LGG: Brain lower grade glioma
LIHC: Liver hepatocellular carcinoma
LUAD: Lung adenocarcinoma
LUSC: Lung squamous cell carcinoma
MESO: Mesothelioma
OV: Ovarian serous cystadenocarcinoma
PAAD: Pancreatic adenocarcinoma
PCPG: Pheochromocytoma and paraganglioma
PRAD: Prostate adenocarcinoma
READ: Rectum adenocarcinoma
SARC: Sarcoma
SKCM: Skin cutaneous melanoma
STAD: Stomach adenocarcinoma
TGCT: Testicular germ cell tumors
THCA: Thyroid carcinoma
THYM: Thymoma
UCEC: Uterine corpus endometrial carcinoma
UCS: Uterine carcinosarcoma
UVM: Uveal melanoma
