# Supplementary material for: An integrative pan-cancer analysis of molecular characteristics and oncogenic role of mitochondrial creatine kinase 1A (CKMT1A) in human tumors
Source: Sci Rep. 2022 Jun 15;12:10025. doi: 10.1038/s41598-022-14346-z (PMC9200842; doi:10.1038/s41598-022-14346-z)
Supplement: Supplementary file 1 — Supplementary Information 1. [file 41598_2022_14346_MOESM1_ESM.pdf]

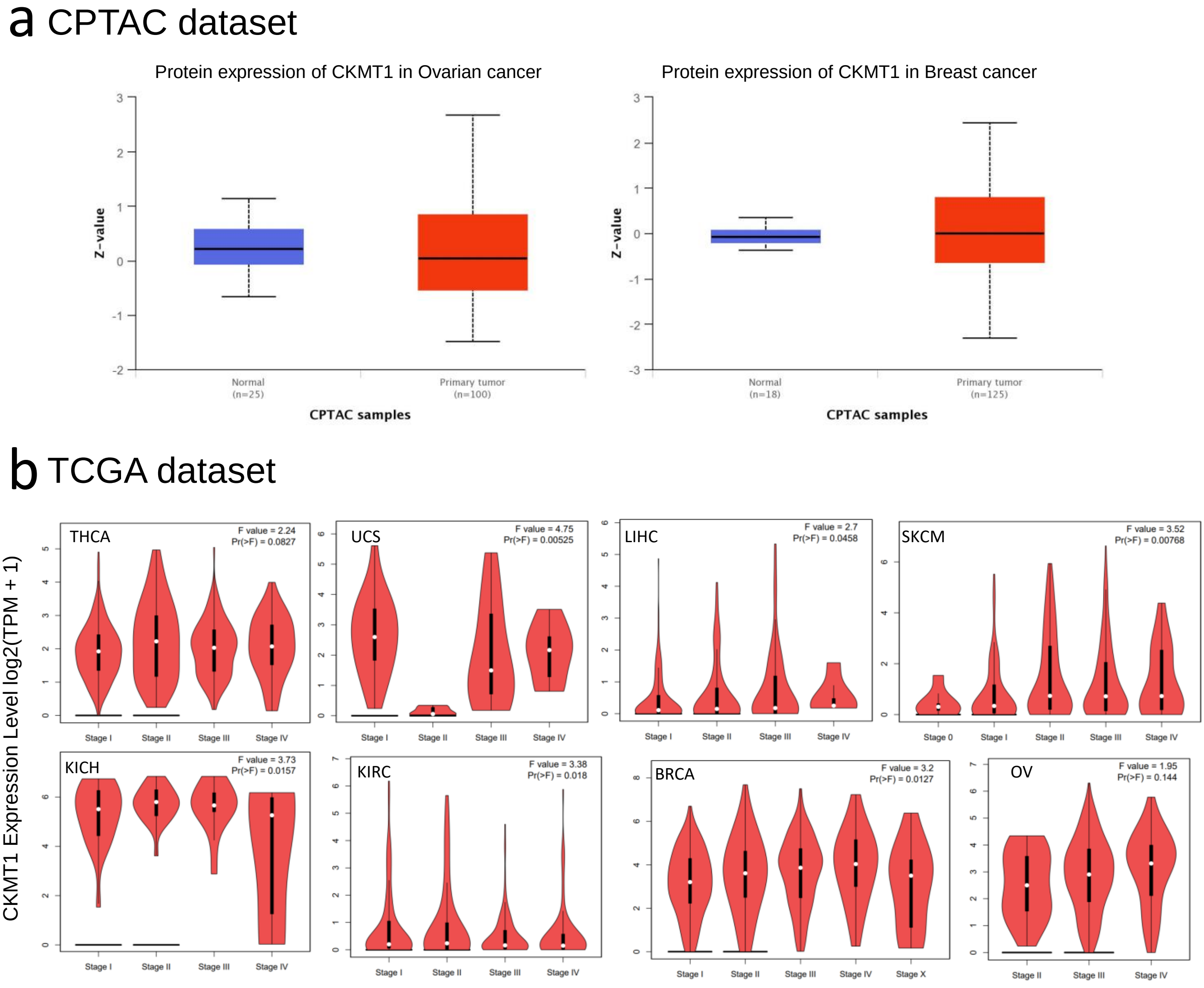

Fig. S1 The CKMT1A Expression level in different tumors and pathological stages. **a** Based on the CPTAC dataset, the expression level of CKMT1A between normal tissue and primary tissue of ovarian cancer and breast cancer were also analyzed. **b** According to the TCGA data, the expression levels of the CKMT1A were analyzed by the main pathological stages (stage I, stage II, stage III, and stage IV) of THCA, UCS, LIHC, SKCM, KICH, KIRC, BRCA and OV. Log2 (TPM+1) was utilized for log-scale.

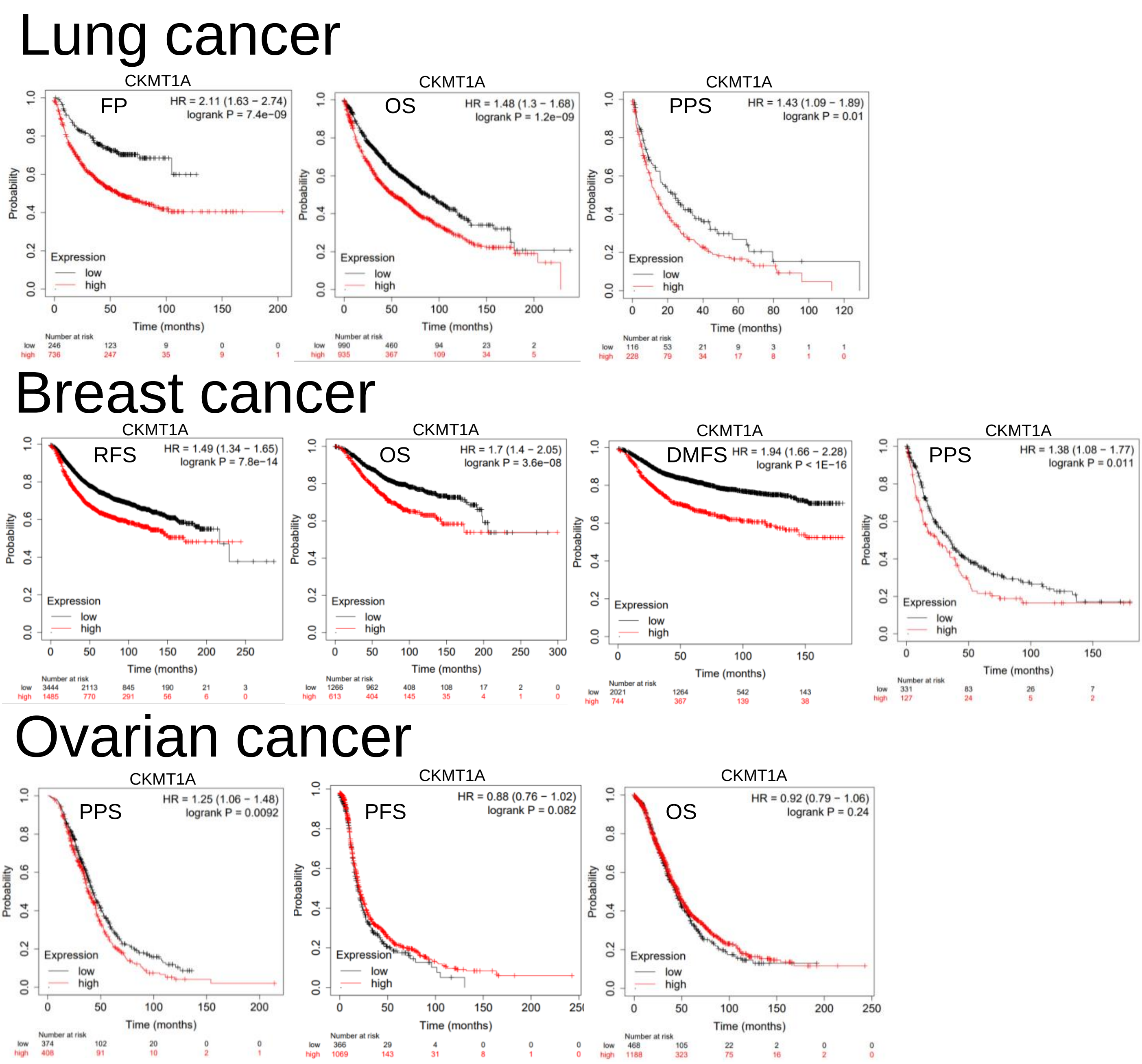

Fig. S2 The Kaplan-Meier curves between the expression level of CKMT1A and survival prognosis in lung cancer, breast cancer and ovarian cancer. FP, first progression; PPS, post-progression survival; RFS, relapse-free survival; DMFS, distant metastasis-free survival; PFS, progress-free survival; DSS, disease-specific survival; OS, overall survival; DFS, disease-free survival.

Gastric cancer

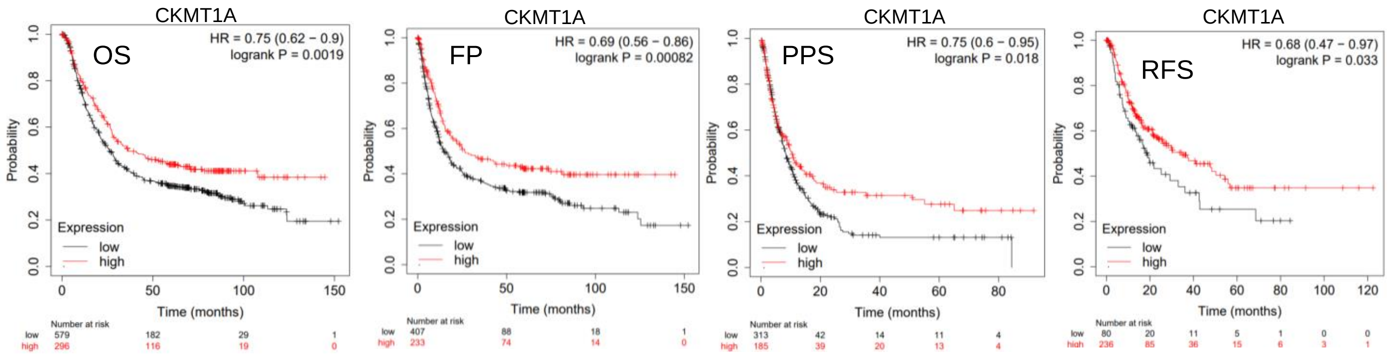

Liver cancer RNA-seq

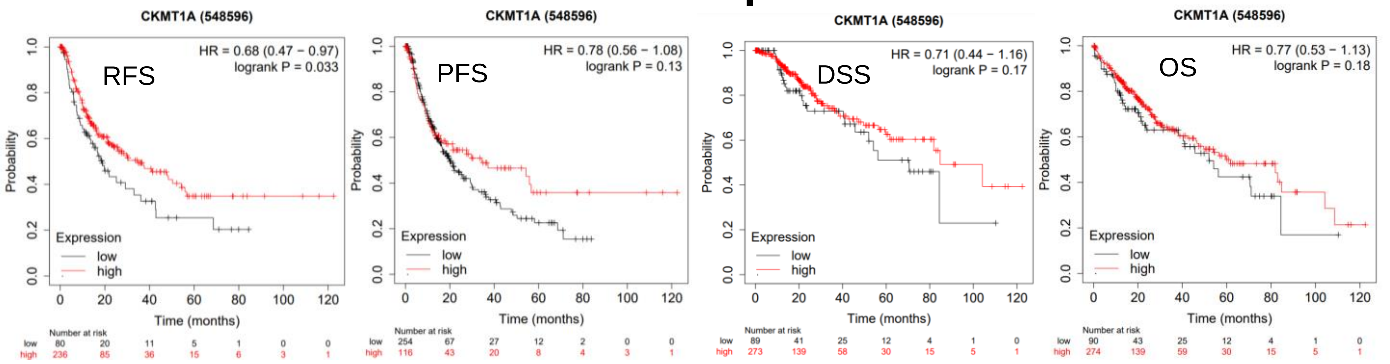

Fig. S3 The Kaplan-Meier curves between the expression level of CKMT1A and survival prognosis in gastric cancer and liver cancer. FP, first progression; PPS, post-progression survival; RFS, relapse-free survival; DMFS, distant metastasis-free survival; PFS, progress-free survival; DSS, disease-specific survival; OS, overall survival; DFS, disease-free survival.

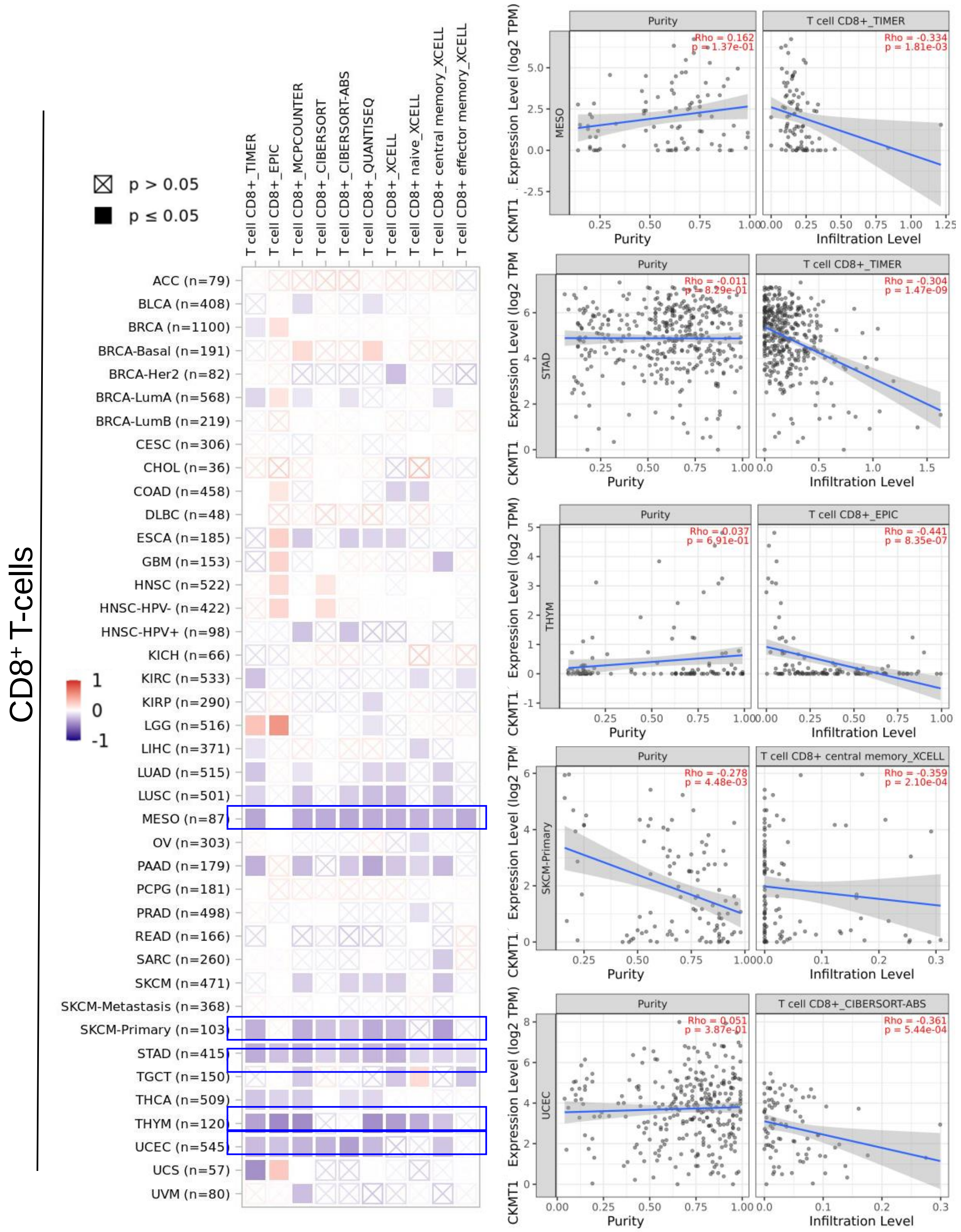

Figure S4. Correlation analysis between the expression of CKMT1A and immune infiltration of $CD8^+$ T-cells. a. The correlation map between CKMT1A expression level and the infiltration level of $CD8^+$ T-cells across all types of cancer in TCGA. b. The relationship of CKMT1A and infiltration level of $CD8^+$ T-cells in BLCA, BRCA, COAD, ESCA, HNSC, LGG, LUAD, PAAD, READ and STAD. Rho, The Value of Spearman's correlation; ACC, Adrenocortical carcinoma; BLCA, Bladder Urothelial Carcinoma; BRCA, Breast invasive carcinoma; CESC, Cervical squamous cell carcinoma and endocervical adenocarcinoma; CHOL, Cholangio carcinoma; COAD, Colon adenocarcinoma; DLBC, Lymphoid Neoplasm Diffuse Large B-cell Lymphoma; ESCA, Esophageal carcinoma; GBM, Glioblastoma multiforme; HNSC, Head and Neck squamous cell carcinoma; KICH, Kidney Chromophobe; KIRC, Kidney renal clear cell carcinoma; KIRP, Kidney renal papillary cell carcinoma; LAML, Acute Myeloid Leukemia; LGG, Brain Lower Grade Glioma; LIHC, Liver hepatocellular carcinoma; LUAD, Lung adenocarcinoma; LUSC, Lung squamous cell carcinoma; MESO, Mesothelioma; OV, Ovarian serous cystadenocarcinoma; PAAD, Pancreatic adenocarcinoma; PCPG, Pheochromocytoma and Paraganglioma; PRAD, Prostate adenocarcinoma; READ, Rectum adenocarcinoma; SARC, Sarcoma; SKCM, Skin Cutaneous Melanoma; STAD, Stomach adenocarcinoma; TGCT, Testicular Germ Cell Tumors; THCA, Thyroid carcinoma; THYM, Thymoma; UCEC, Uterine Corpus Endometrial Carcinoma; UCS, Uterine Carcinosarcoma; UVM, Uveal Melanoma.
