# Supplementary material for: An integrative pan-cancer analysis of molecular characteristics and oncogenic role of mitochondrial creatine kinase 1A (CKMT1A) in human tumors
Source: Sci Rep. 2022 Jun 15;12:10025. doi: 10.1038/s41598-022-14346-z (PMC9200842; doi:10.1038/s41598-022-14346-z)
Supplement: Supplementary file 2 — Supplementary Information 2. [file 41598_2022_14346_MOESM2_ESM.docx]

| 50 CKMT1A-interacting proteins | STRC NME3 PFKM PFKL NME4 RASEF CCL16 GGT7 APP AK1 ALPPL2 ALDOA GOT2 ALPI HIBADH FH LDHA ENO4 GAPDHS GAMT NME2 PPIF LDHC AK2 PPIP5K1 LDHAL6B CATSPER2 GOT1 LDHB ALPP AK9 PYGL CKMT1B PYGB ASB9 NME1-NME2 ACO2 GGT6 TPI1 PYGM GGT5 ALDOC SLC6A8 SLC25A5 CS HKDC1 GAPDH RAB28 LDHAL6A GATM |
| --- | --- |
| 100 CKMT1A correlated genes | VDR EPS8L3 UCHL3 FAM83B PKP3 FAM83F AIM1L STK24 GSKIP MAPK13 NDFIP2 FUT3 C1orf106 PAK6 RP11-304L19.3 SH3RF2 TMPRSS4 RHOV MAPK6 PTK6 HR ABHD17C MYO1A MST1R CYP2S1 PVRL1 IRF6 RP11-304L19.1 FAM83E RAPGEFL1 ATP10B MKI67 CEP55 BRI3BP GJB5 ZDHHC13 ADAP1 PGAM5 ETHE1 ATP8B1 PITX1 TUBA4A TRIM29 CYCS MISP DTX2 CDCP1 PTBP3 RP11-44F14.2 SMAGP MRRF ZC3H12A DQX1 TRIM7 GJB3 RAB25 RRM2 CBLC SERPINB5 FUT2 RP11-44F14.8 KLF5 LAD1 TTC22 PKP1 ZDHHC3 ST14 MYH14 PLS1 C6orf132 PERP REEP4 TMEM54 ARHGEF5 DSG3 OVOL1 FERMT1 CKMT1B SH2D3A TUBA1C CAPN1 GGT6 FAM83G PLCB3 S100A16 ZNF57 NIPAL1 ECT2 ANKRD22 SLC25A5 POF1B FAM83H ATP1B3 ETS2 RP3-523K23.2 RHBDL2 SDCBP2 KLF3 NRARP PLEKHG6 |

The table of CKMT1A-interacting proteins and correlated genes

**Additional file 2.** The list of the top 100 CKMT1A-correlated genes in GEPIA2 and 50 potential CKMT1A-interacting proteins in the STRING tool.
